# Health Trajectories in Swedish Centenarians

**DOI:** 10.1093/gerona/glaa152

**Published:** 2020-06-22

**Authors:** Davide L Vetrano, Giulia Grande, Alessandra Marengoni, Amaia Calderón-Larrañaga, Debora Rizzuto

**Affiliations:** 1 Aging Research Center, Department of Neurobiology, Care Sciences and Society, Karolinska Institutet and Stockholm University, Stockholm, Sweden; 2 Centro Medicina dell’Invecchiamento, IRCCS Fondazione Policlinico Universitario “A. Gemelli,” and Università Cattolica del Sacro Cuore, Rome, Italy; 3 Department of Clinical and Experimental Science, University of Brescia, Italy; 4 Stockholm Gerontology Research Center, Stockholm, Sweden

**Keywords:** Centenarians, Disability, Cognitive impairment, Multimorbidity

## Abstract

**Background:**

Longitudinal studies describing centenarians’ health trajectories are currently lacking. We compared health trajectories of older adults becoming centenarians and their shorter-living counterparts in terms of chronic diseases, disability, and cognitive decline.

**Methods:**

We identified 3,573 individuals participating in the Kungsholmen Project and the Swedish National Study on Aging and Care in Kungsholmen who lived <100 years and 222 who survived to their 100th birthday. Trajectories of chronic diseases, disability (impaired activities of daily living), and cognitive status were obtained via linear mixed models over 13 years.

**Results:**

Centenarians had fewer chronic diseases than noncentenarians. Before age 85, centenarians showed slower health changes. In centenarians, multimorbidity, disability, and cognitive impairment occurred 4 to 9 years later than in noncentenarians. After age 85, the speed of accumulation of chronic diseases, disabilities, and cognitive decline accelerated in centenarians. At age 100, 39% of the centenarians were cognitively intact and 55% had escaped disability. Only 5% were free of multimorbidity at age 100. When compared with their shorter lived counterparts, in terms of years spent in poor health, centenarians experienced more years with multimorbidity (9.4 vs 6.8 years; *p* < .001), disability (4.3 vs 3.1 years; *p* = .005), and cognitive impairment (6.3 vs 4.3 years; *p* < .001).

**Conclusions:**

Older people who become centenarians present a delay in the onset of morbidity, but spend more years in this condition compared to their shorter lived peers. The observation of older adults’ health trajectories might help to forecast healthier aging, and plan future medical and social care delivery.

The global increase in life expectancy has led to an unprecedented rise in the proportion of very old people (1). For instance, in Sweden, there were only 105 centenarians in 1970; a number that increased 20-fold by 2017, reaching a total of 2,084 (2). The aging of the population is one of humankind’s major accomplishments, but ensuring that people age healthily remains a challenging goal. The first concern is related to the quality of life—usually declining with age—of seniors who live through their 70th decade and beyond (3). The second issue relates to the future demands of medical and social care, which will greatly depend on the health status of the coming generations of old and oldest old individuals. The comparison of centenarians’ health status with that of their shorter living counterparts may enable us to better understand the ongoing demographic transition and to detect—at the individual level—the ages at which health transitions are happening, both of which could improve prognostication.

According to Fries and colleagues the burden of illness and disability will be compressed into a shorter period during the last years of life as a consequence of the demographic transition in which we are living. This can be attributed to improvements in biomedical sciences and public health, which will delay and reduce the onset of several noncommunicable diseases (4,5). On the other hand, it is unclear whether morbidity will actually be postponed at a faster pace than improvements in life expectancy (6–8). Recent studies on centenarians—probably the best models of long-term survival—found that although some live with disease and diminished functioning, others succeed in remaining healthy and escaping functional impairments (9–18). However, the majority of studies concerning centenarians have been based on cross-sectional measurements of health and functioning (9–13,15–17). Moreover, many of these studies excluded institutionalized individuals, severely biasing the estimation of the frequency of illnesses and functional dependence within this age group. A better understanding of the health trajectories of those becoming centenarians requires longitudinal observation at the individual level and this should include periods both before and after reaching 100 years. To the best of our knowledge, no previous studies have quantified the gap between centenarians and their shorter living counterparts in terms of healthy lived life-years.

The aims of this study were to compare the trajectories of chronic disease, disability, and cognitive function of older adults who will become centenarians with those of their shorter lived counterparts.

## Method

### Study Population

Data were gathered from two population-based longitudinal studies, both carried out in the Kungsholmen district in central Stockholm, Sweden, namely the Kungsholmen Project (KP, 1987–2000) (19) and the Swedish National study on Aging and Care in Kungsholmen (SNAC-K, 2001–2013) (20). In both studies, all eligible residents living either at home or in institutions, in Kungsholmen and adjacent areas, were asked to participate.

The KP targeted all inhabitants of the Kungsholmen district aged 75 years and older (range 75–102 years) in October 1987 (born before 1912). Of the eligible people (*n* = 2,368), 1,810 (76.4%) agreed to participate in the baseline survey (1987–1989). Health assessments were conducted at approximately 3-year intervals until 2000, and the survival status of the participants was assessed until 2013.

SNAC-K is an ongoing longitudinal study that includes a random sample of people aged 60+ years (range 60–104 years) from 11 age cohorts. To compensate for the potential attrition at follow-up, the two youngest and the four oldest age groups were oversampled. The current study includes data from baseline (2001−2004) and three follow-ups, up to 2013. At baseline (2001−2004), 3,363 (73.3%) of all eligible persons (*n* = 4,590) were examined, and their health and survival statuses were assessed until 2013 and 2018, respectively.

Participants were included in the analytic sample if they survived to 100 years by the end of the follow-up or by the date data were available (ie, 2013 for KP and 2018 for SNAC-K), or if they died before turning 100 years in the same period. By the end of the follow-up period, 222 centenarians were identified. Of them, three had missing data for activities of daily living (ADL; 1.4%). The age of all participants was confirmed by their unique Swedish national identification number (21).

### Data Collection

#### Procedures

In all waves of KP and SNAC-K, data were collected at the Aging Research Center, Karolinska Institutet, Stockholm, in accordance with standard procedures. Trained staff performed: face-to-face interviews; clinical, functional, and laboratory examinations; and cognitive tests. Home visits were carried out for those who agreed to participate but were unable to visit the research center. The same protocols were used at baseline and follow-ups to collect information about each person and their context, and similar protocols were used for the data collection in both KP and SNAC-K (19,20). Participants’ proxies intervened in reporting the requested information in 18% of cases. All participants, or proxies in the case of cognitively impaired persons, provided written informed consent. The Regional Ethical Review Board in Stockholm, Sweden, approved the protocols of the KP and SNAC-K studies.

#### Health indicators

We examined three dimensions of health—chronic diseases, disability, and cognitive status—that were assessed at baseline and during follow-up examinations. *Chronic diseases* were diagnosed or ascertained by physicians on the basis of clinical examination, medical history, laboratory data, current drug use, and linkage to the outpatient and inpatient registers. Chronic diseases were classified in accordance with the International Classification of Diseases 10th Revision (ICD-10). A disease was defined as chronic if it was of prolonged duration and either (a) left residual disability or worsened quality of life or (b) required a long period of care, treatment, or rehabilitation (22). A total of 32 chronic conditions were considered in the present study and further clustered into 10 groups of conditions: anemia, cardiovascular, digestive, endocrine, malignancy, neuropsychiatric, musculoskeletal, neurosensorial, respiratory, and urological chronic diseases (Supplemental Table S1) (23,24). Disease status was operationalized in the analyses as no chronic disease and having one or more chronic diseases from the group of conditions. The baseline prevalence of each condition by survival profile (centenarians vs noncentenarians) is reported in Supplemental Table S3. Multimorbidity was defined as the presence of two or more chronic diseases in one individual. *Disability* was measured through number of impairments (dependence) in the Katz’s ADL (scoring 0–6) scale, which includes the following activities: bathing, dressing, toileting, transferring, continence, and eating. *Cognitive function* was assessed through the Mini-Mental State Examination (MMSE), a 30-point questionnaire that includes items related to different cognitive functions such as: orientation to time and place, attention and calculation, recall, language, ability to follow written and verbal commands, and visual construction (25). A cutoff score of 25 was used to define cognitive impairment.

#### Survival and health profile

Participants were classified as noncentenarians (those who died before reaching the age of 100 years, *n* = 3,573) and centenarians (those who reached 100 years, *n* = 222). In addition, among centenarians we identified two subgroups according to each of the three health indicators. Centenarians were considered *healthy* if they had no more than one chronic disease and *unhealthy* if they had two or more chronic diseases; *healthy* if they had no more than one ADL impairment and *unhealthy* if they had two or more ADL impairments; and *healthy* if they had an MMSE score greater than or equal to 25 and *unhealthy* if they had an MMSE score below 25 by the time they reached 100 years or during the last assessment.

#### Sociodemographic characteristics

In terms of sociodemographic characteristics, sex and education level were assessed at baseline, and age and living situation were documented during all follow-up examinations. Educational attainment was categorized as: (i) elementary school (Grade 1–9), (ii) high school (Grade 10–12), or (iii) university or above. Information on housing type was categorized as: (i) living in nursing homes or (ii) living in all other types of housing (house, apartment, supported housing facility, and other). Marital status was categorized as: (i) married, (ii) single, or (iii) widowed or divorced. Cohabitation status was categorized as: (i) living alone or (ii) living with someone. Finally, information on smoking habits (current vs former/never) and alcohol use was collected.

### Statistical Analyses

Health status before death of noncentenarians was compared with that of centenarians at study entry. Linear mixed-effects models were used to estimate the trajectories of the health indicators using repeated measurements over 13 years, and models were adjusted for potential confounders (sex, education level, smoking habit, alcohol consumption, birth cohort, and study cohort). Trajectories were built, centering the covariates on their means; they can thus be interpreted as the trajectory of an individual with average characteristics within each survivorship group. The age of the participants was used as the time scale, and the two-way interaction between follow-up time and survival profile was added in each model. The intercept and follow-up time provided the fixed and random effects. Unstructured covariance between both random-effect parameters was assumed, and potential nonlinear relationships between the three outcomes (number of chronic diseases, disability, and cognitive impairment) and survivorship status (centenarians vs noncentenarians) were investigated using a quadratic transformation of the follow-up time (age). Departure from linearity was assessed by testing the null hypothesis that the coefficient of the quadratic term was equal to zero. The interaction between survival profile (centenarians vs noncentenarians) and birth cohort was also assessed. To investigate the differences between centenarians and their counterparts in terms of years lived with chronic diseases, disability, and cognitive impairment, a two-tail ANOVA was used to compare the time from the onset of such conditions (including when these were present at baseline) to death across the two groups. Finally, heterogeneity among centenarians was assessed by estimating the age-adjusted prevalence of each of the three health indicators (healthy vs unhealthy) across the two survival profiles. When the information at exactly the age of 100 years was not available, the nearest (±3 years) available assessment was considered. All analyses were performed using Stata version 15 (StataCorp, TX, USA).

## Results

By the end of the observation period, 222 centenarians had been identified. About 90% of them were woman and 8% had higher education. At study entry, participants who became centenarians were older than their counterparts (mean age 90 ± 7 vs 81 ± 8 years; *p* = .001). Moreover, centenarians were less educated, more frequently widowed or single, less likely to be institutionalized, and more cognitively impaired than noncentenarians (Table 1). Supplemental Table S2 reports the baseline characteristics of centenarians and noncentenarians after excluding participants <73 years old from the noncentenarian group, and those >100 from the centenarian group.

**Table 1.** Baseline Characteristics of Centenarians and Noncentenarians

	Noncentenarians (*N* = 3,573)	Centenarians (*N* = 222)	*P* value
Age	81.3 ± 8.2	90.1 ± 7.2	.001
Female sex	69.7%	91.0%	<.001
Education level			
Elementary	38.0%	39.4%	.027
High school	47.1%	52.3%	
University or above	14.9%	8.3%	
Marital status			
Single	18.8%	24.8%	<.001
Widowed/divorced	55.4%	67.1%	
Married/partnered	25.8%	8.1%	
Living situation			
Alone	60.5%	70.7%	<.001
With someone	16.4%	22.1%	
In institution	23.1%	7.2%	
Number of chronic diseases	3.5 ± 2.2	3.6 ± 2.2	.709
Number of impaired ADLs	0.7 ± 1.4	0.9 ± 1.6	.074
MMSE	25.2 ± 6.6	24.2 ± 7.6	.027
Survival time	7.2 ± 4.9	9.7 ± 6.2	<.001

*Notes:* Numbers are means ± standard deviations for continuous variables and percentages for categorical variables. Number of people with missing data: three for ADL (1.4%) and one for social care use (0.5%). ADL, activities of daily living; MMSE, Mini-Mental State Examination.

**Figure 1.**
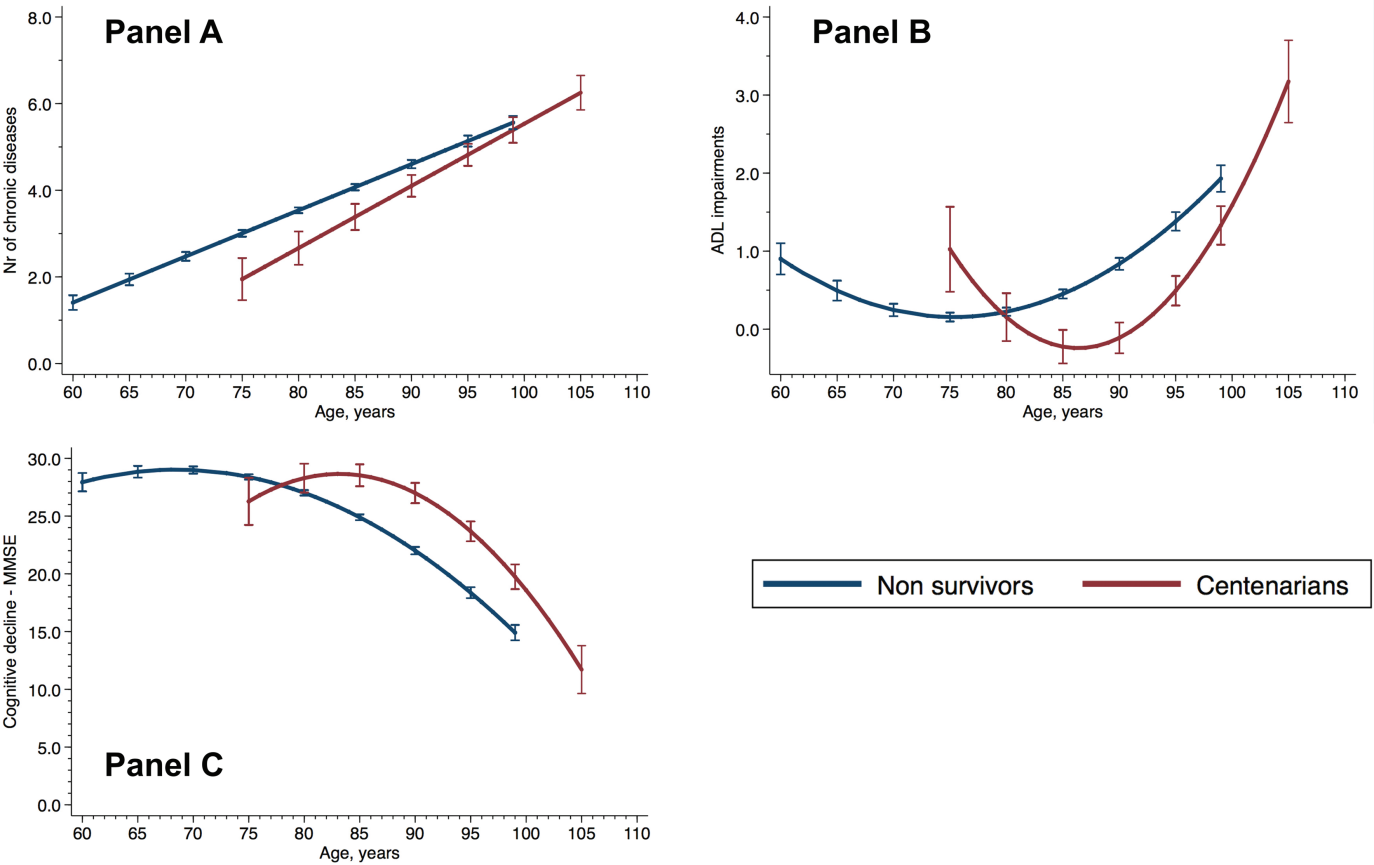
Trajectories (with 95% confidence intervals) of chronic diseases (**A**), ADL impairments (**B**), and cognitive decline (MMSE, **C**) across age in centenarians and noncentenarians. Bars represent 95% CI. ADL, activities of daily living; MMSE, Mini-Mental State Examination. Three people with missing data for ADL (1.4%).

Figure 1 displays trajectories of chronic diseases, ADL impairments, and MMSE scores across age in years for centenarians and noncentenarians, based on a total of 18,975 repeated assessments carried out in SNAC-K and KP during the study period. A significant nonlinear relationship was found between surviving to 100 years and trajectories of ADL impairments and MMSE over time (nonlinearity test *p* < .001 for both). On average, centenarians had fewer chronic diseases than noncentenarians before their ninth decade of life, as suggested by nonoverlapping CIs. However, given the faster accumulation of chronic diseases experienced by centenarians (*p* for linear interaction with time = .004), this difference became nonsignificant after 95 years. Before the age of 85 years, centenarians demonstrated slower changes in disability (*p* for quadratic interaction with time <.001) status and cognitive function in comparison to noncentenarians (*p* for quadratic interaction with time = .02). However, after the age of 85 years, an acceleration (ie, stronger interaction with time) in the accumulation of ADL impairments and cognitive decline was observed. In terms of physical and cognitive function, the decade between 75 and 85 years was the period when trajectories started to diverge between the two groups. On average, while noncentenarians were observed to suffer from three or more diseases at the age of 75 centenarians did so at the age of 83, 8 years later. Moreover, noncentenarians presented with one or more impaired ADL at the age of 92 and centenarians at the age of 98, 6 years later. Finally, while noncentenarians scored on average below 25 on the MMSE at age 84, centenarians did so 9 years later at age 93. Estimated means and 95% CI of the health indicators for individuals with average characteristics are reported in Supplemental Table S4 for different ages.

When testing two-way interactions between survival profile and birth cohort, we found that younger cohorts experienced a slower decline in ADL performance (*β* for interaction −0.02 for every 10-year increase in birth cohort, 95% CI −0.05; −0.01) but a faster decline in cognitive function (*β* for interaction 0.22 for every 10-year increase in birth cohort, 95% CI 0.11; 0.33).

Table 2 shows the age-adjusted prevalence of ADL impairments, chronic diseases, and MMSE categories of centenarians at the time of their follow-up when they were closest to age 100 (age range 97−103 years). The prevalence of ADL impairments ranged from 25% to 44%. The most common chronic conditions among centenarians were cardiovascular diseases (88%), and the least common were respiratory diseases (12%). At this time point, 55.4% presented a MMSE score higher than 20.

**Table 2.** Age-Adjusted Prevalence of Chronic Diseases and Activities of Daily Living (ADL) Impairments at the Nearest Assessment to 100 Years (*N* = 222; age range 97−103 years)

Health Characteristics	ADL Impairments (%)	Chronic Diseases (%)	MMSE (%)
Bathing	44.0		
Continence	34.2		
Dressing	383		
Eating	28.4		
Toileting	29.2		
Transferring	25.1		
Anemia		45.8	
Cardiovascular diseases		88.0	
Digestive disorders		41.0	
Endocrine diseases		25.3	
Malignancy		13.3	
Neuropsychiatric diseases		59.0	
Musculoskeletal diseases		63.9	
Neurosensorial diseases		65.1	
Respiratory diseases		12.0	
Urological disorders		54.2	
MMSE 0–10			18.1
MMSE 11–20			26.5
MMSE 21–30			55.4

Three people with missing data for ADL (1.4%).

ADL, Activities of Daily Living; MMSE, Mini-Mental State Examination.

When we compared centenarians with their shorter lived counterparts in terms of years spent with poor health, we found that centenarians the formers spent more years with multimorbidity (9.4 vs 6.8 years; *p* < .001), disability (4.3 vs 3.1 years; *p* = .005), and cognitive impairment (6.3 vs 4.3 years; *p* < .001).

**Figure 2.**
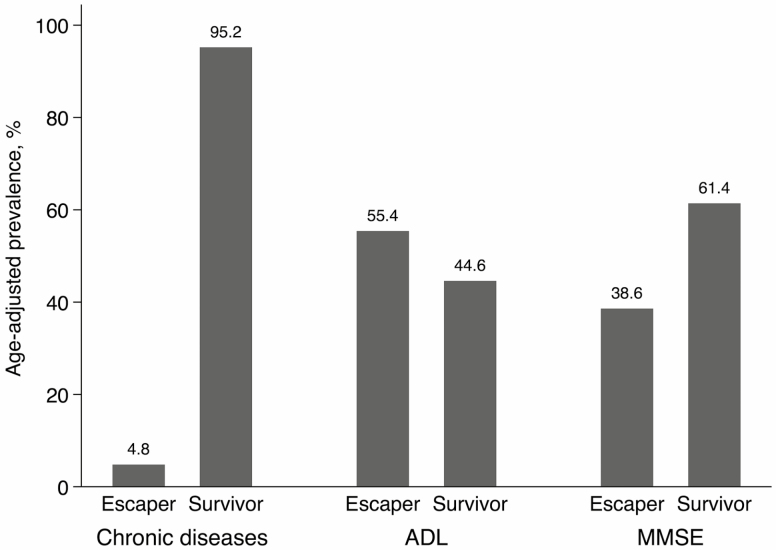
Age-adjusted prevalence of healthy and unhealthy centenarians according to the number of chronic diseases, ADL impairments, and cognitive performance at the nearest moment to reaching 100 years (age range 97−103 years). ADL, activities of daily living; MMSE, Mini-Mental State Examination. Healthy centenarians are defined as having fewer than two chronic diseases, fewer than two ADL impairments, or an MMSE score above or equal to 25. Unhealthy centenarians are defined as having more than one chronic disease, more than one ADL disability, or an MMSE score below 25.

Although centenarians were generally healthier than noncentenarians, heterogeneity exists in this group’s health profile. Figure 2 presents the age-adjusted distribution of healthy and unhealthy centenarians for each of the three health indicators. Only 5% of centenarians reached 100 years with fewer than two chronic diseases. Approximately 55% of centenarians escaped disability (fewer than two ADL impairments) and 39% remained cognitively intact (MMSE ≥ 25). In summary, 33% of centenarians could be considered healthy for at least two out of three health indicators, but only 1.2% were healthy for all three of them.

## Discussion

Findings from this longitudinal study on health trajectories of Swedish centenarians suggest that persons reaching their 100s have more favorable health profiles in the previous decades than their counterparts who die earlier. In our sample population, centenarians developed multimorbidity, disability, and cognitive impairments 8, 6, and 9 years later than noncentenarians, respectively. In spite of the slow progression of chronic diseases and functional impairment before 85 years, centenarians experience an acceleration in health status decline after this age. At the same time, they seem to spend more years with multimorbidity, disability and cognitive impairment. To the best of our knowledge, this is the first study employing long-term, repeated observations to draw centenarians’ health trajectories and to quantify the health gap between them and their shorter living counterparts.

Previous studies found that individuals surviving to very old ages experience a postponement in morbidity toward their very last years of life (26). Results from the Longevity Genes Project and the New England Centenarian Study (27) indicate that long-lived individuals have a delayed age of onset for a number of chronic diseases such as cancer, cardiovascular diseases, hypertension, and diabetes mellitus, each of which are well-known determinants of dependency (28). Beyond chronic diseases, and in line with our results, Anderson and colleagues also observed a progressive delay in functional and cognitive impairment in centenarians and supercentenarians (those living beyond the age of 110) (26). Similar results were reported by Engberg and colleagues (29). Finally, according to a Danish cohort study by Engberg and colleagues, centenarians experience less hospitalizations and shorter lengths of hospital stays than their shorter lived counterparts (30). Ours is the first study to quantify the time gap between centenarians and their shorter lived counterparts of the same age to reach equal levels of chronic disease burden and functional impairment. By following health trajectories of people living until their 100s, we found that they reach the same level of multimorbidity (three or more diseases) and disabilities 8 and 6 years later in life, respectively, compared to noncentenarians, and cognitive impairment up to 9 years later. Despite the morbidity postponement observed in centenarians, our results demonstrate that this group spends one to two additional years with multimorbidity, disability, and cognitive impairment, when compared to their shorter living counterparts (4). Given the long follow-up available in our study, it was possible to trace trajectories of chronic diseases, disability, and cognition in centenarians across a long period of time. These trajectories indicate that health changes between 75 and 85 years are particularly important in terms of longevity prediction. In turn, the observation of health changes during specific sensitive decades of life might be helpful to timely identify exceptionally long-living persons. Studying the predictors of longevity could be important both for planning healthcare resources and to design observational studies on longevity patterns and associated factors.

We found a considerable heterogeneity in the health status of centenarians. Only 1% of centenarians were free from multimorbidity, cognitive decline, and disability, but 33% were free from two of these three indicators. Health diversity among centenarians is well known in the literature; our study added to this heterogeneity by including participants living in nursing homes. While some centenarians escape or delay chronic diseases and functional and cognitive impairment, around half of them experience aging trajectories that are similar to noncentenarians (31). We found that only 5% of survivors reached 100 years with fewer than two chronic diseases. This figure is lower than what has been previously reported (14), which might be due to the accurate disease assessment and recording carried out in both KP and SNAC-K as well as the fact that we also included people living in institutions. Still, suffering from multimorbidity was not necessarily associated with disability. In fact, the prevalence of functional independence in our study (approximately half of our sample) was similar to that reported previously (32). This supports the need for tertiary prevention even in very advanced ages. However, findings from studies on cognitive impairment in centenarians are difficult to compare due to differences in their assessment methods. It is worth noting that 38% of our sample was cognitively intact, confirming that dementia is not inevitable in the oldest old.

This study has several limitations. First, due to the large prevalence of women among those who survived to their 100s, we were unable to analyze sex differences. Second, we cannot discard that some of the differences between health trajectories of centenarians and noncentenarians observed during their seventh and eighth decades are explained by a longitudinal differential selection that penalizes the sickest noncentenarians. Third, not accounting for the severity of single chronic diseases might have led to an overestimation of the disease burden. However, the co-occurrence of numerous chronic diseases with overlapping signs and symptoms makes it very challenging to stage the severity of single chronic diseases in multimorbid centenarians. Finally, caution should be used when interpreting centenarians’ health trajectories at younger ages (Figure 1), given the small number of available observations available for centenarians at younger ages. One strength of the study is the longitudinal design and the long follow-up. Another strength is the assessment of chronic diseases and function by physicians and nurses. Older persons living in institutions were included, increasing the reliability of our estimations at the population level.

In conclusion, according to this population-based longitudinal study, older people who will eventually become centenarians experience more favorable health trajectories, compared to their noncentenarian counterparts of the same age, during their 70s, 80s, and 90s. For centenarians participating in our study, multimorbidity, disability, and cognitive impairment seem to be postponed by 8, 6, and 9 years, respectively, in comparison to noncentenarians. At the same time, they experience an expansion of morbidity, which is exhibited through the higher number of years spent in poor health, compared to noncentenarians. Longevity seems to be accompanied by longer life spent with chronic diseases and functional impairment, which might trigger more medical and care needs.

## Funding

This work was supported by the funders of the Swedish National study on Aging and Care; the Ministry of Health and Social Affairs, Sweden; the participating County Councils and Municipalities; the Swedish Research Council; and Karolinska Institutet (KID-funding), Stockholm, Sweden. Swedish Research Council for Health, Working Life and Welfare. The funders had no role in study design, data collection and analysis, decision to publish, or preparation of the manuscript.

## Author Contributions

DLV, GG, and DR contributed to the conception and design of the study. DR conducted the statistical analyses. DLV and GG conducted the literature search. All the authors contributed to interpretation of the results. DLV, DR, GG, and AM drafted the first version of the manuscript. All the authors critically revised the manuscript for important intellectual content. All the authors made a significant contribution to the research and the development of the manuscript and approved the final version for publication. The K-P and SNAC-K personnel collected the data for the study.

## Conflicts of Interest

None reported.

## Supplementary Material

glaa152_suppl_Supplementary_Tables-and-Figures

## References

[1] Christensen K, Doblhammer G, Rau R, Vaupel JW Ageing populations: the challenges ahead. Lancet. 2009;374:1196–1208. doi: 10.1016/S0140-6736(09)61460-4 19801098 PMC2810516

[2] Sweden Si. Accessed November 1, 2019. http://www.scb.se/Sweden2018

[3] Crimmins EM, Beltran-Sanchez H Mortality and morbidity trends: is there compression of morbidity? J Gerontol B Psychol Sci Soc Sci. 2011;66(1):75–86. doi: 10.1093/geronb/gbq088. 21135070 PMC3001754

[4] Fries JF Aging, natural death, and the compression of morbidity. N Engl J Med. 1980;303:130–135. doi: 10.1056/NEJM198007173030304 7383070

[5] Olshansky SJ, Ault AB The fourth stage of the epidemiologic transition: the age of delayed degenerative diseases. Milbank Q. 1986;64: 355–391. 3762504

[6] Robine JM, Michel JP Looking forward to a general theory on population aging. J Gerontol A Biol Sci Med Sci. 2004;59:M590–M597. doi: 10.1093/gerona/59.6.m590 15215269

[7] Guralnik JM Robine and Michel’s “Looking forward to a general theory on population aging”: population aging across time and cultures: can we move from theory to evidence? J Gerontol A Biol Sci Med Sci. 2004;59:M606–M608; author reply M616. doi: 10.1093/gerona/59.6.m606 15215277

[8] Melzer D, Lan TY, Tom BD, Deeg DJ, Guralnik JM Variation in thresholds for reporting mobility disability between national population subgroups and studies. J Gerontol A Biol Sci Med Sci. 2004;59:1295–1303. doi: 10.1093/gerona/59.12.1295 15699529

[9] Andersen-Ranberg K, Schroll M, Jeune B Healthy centenarians do not exist, but autonomous centenarians do: a population-based study of morbidity among Danish centenarians. J Am Geriatr Soc. 2001;49:900–908. doi: 10.1046/j.1532-5415.2001.49180.x 11527481

[10] Andersen-Ranberg K, Christensen K, Jeune B, Skytthe A, Vasegaard L, Vaupel JW Declining physical abilities with age: a cross-sectional study of older twins and centenarians in Denmark. Age Ageing. 1999;28:373–377. doi: 10.1093/ageing/28.4.373 10459791

[11] Andersen-Ranberg K, Vasegaard L, Jeune B Dementia is not inevitable: a population-based study of Danish centenarians. J Gerontol B Psychol Sci Soc Sci. 2001;56:P152–P159. doi: 10.1093/geronb/56.3.p152 11316833

[12] Gondo Y, Hirose N, Arai Y, Inagaki H, Masui Y, Yamamura K, et al. Functional status of centenarians in Tokyo, Japan: developing better phenotypes of exceptional longevity. J Gerontol A Biol Sci Med Sci. 2006;61(3):305–310. doi: 10.1093/gerona/61.3.305 16567382

[13] Silver MH, Jilinskaia E, Perls TT Cognitive functional status of age-confirmed centenarians in a population-based study. J Gerontol B Psychol Sci Soc Sci. 2001;56:P134–P140. doi: 10.1093/geronb/56.3.p134 11316831

[14] Evert J, Lawler E, Bogan H, Perls T Morbidity profiles of centenarians: survivors, delayers, and escapers. J Gerontol A Biol Sci Med Sci. 2003;58:232–237. doi: 10.1093/gerona/58.3.m232 12634289

[15] Franceschi C, Motta L, Valensin S, Rapisarda R, Franzone A, Berardelli M, et al. Do men and women follow different trajectories to reach extreme longevity? Italian Multicenter Study on Centenarians (IMUSCE). Aging (Milano). 2000;12(2):77–84. doi: 10.1007/BF03339894 10902049

[16] Davey A, Elias MF, Siegler IC, Lele U, Martin P, Johnson MA, et al. Cognitive function, physical performance, health, and disease: norms from the georgia centenarian study. Exp Aging Res. 2010;36(4):394–425. doi: 10.1080/0361073X.2010.509010 20845120 PMC2941913

[17] Terry DF, Sebastiani P, Andersen SL, Perls TT Disentangling the roles of disability and morbidity in survival to exceptional old age. Arch Intern Med. 2008;168:277–283. doi: 10.1001/archinternmed.2007.75 18268168 PMC2895331

[18] Hitt R, Young-Xu Y, Silver M, Perls T Centenarians: the older you get, the healthier you have been. Lancet. 1999;354:652. doi: 10.1016/S0140-6736(99)01987-X 10466675

[19] Fratiglioni L, Viitanen M, Backman L, Sandman PO, Winblad B Occurrence of dementia in advanced age: the study design of the Kungsholmen Project. Neuroepidemiology. 1992;11 Suppl 1:29–36. doi: 10.1159/000110958 1603245

[20] Lagergren M, Fratiglioni L, Hallberg IR, Berglund J, Elmstahl S, Hagberg B, et al. A longitudinal study integrating population, care and social services data. The Swedish National study on Aging and Care (SNAC). Aging Clin Exp Res. 2004;16(2):158–168. doi: 10.1007/bf03324546 15195992

[21] Ludvigsson JF, Otterblad-Olausson P, Pettersson BU, Ekbom A The Swedish personal identity number: possibilities and pitfalls in healthcare and medical research. Eur J Epidemiol. 2009;24:659–667. doi: 10.1007/s10654-009-9350-y 19504049 PMC2773709

[22] Calderon-Larranaga A, Vetrano DL, Onder G, Gimeno-Feliu LA, Coscollar-Santaliestra C, Carfi A, et al Assessing and measuring chronic multimorbidity in the older population: a proposal for its operationalization. J Gerontol A Biol Sci Med Sci. 2017;72(10):1417–1423. doi: 10.1093/gerona/glw233 28003375 PMC5861938

[23] Rizzuto D, Melis RJF, Angleman S, Qiu C, Marengoni A Effect of chronic diseases and multimorbidity on survival and functioning in elderly adults. J Am Geriatr Soc. 2017;65(5):1056–1060. doi: 10.1111/jgs.14868 28306158

[24] Marengoni A, Rizzuto D, Wang HX, Winblad B, Fratiglioni L Patterns of chronic multimorbidity in the elderly population. J Am Geriatr Soc. 2009;57(2):225–230. doi: 10.1371/journal.pone.0227252 19207138

[25] Folstein MF, Folstein SE, McHugh PR “Mini-mental state.” A practical method for grading the cognitive state of patients for the clinician. J Psychiatr Res. 1975;12(3):189–198. doi: 10.1016/0022-3956(75)90026-6 1202204

[26] Andersen SL, Sebastiani P, Dworkis DA, Feldman L, Perls TT Health span approximates life span among many supercentenarians: compression of morbidity at the approximate limit of life span. J Gerontol A Biol Sci Med Sci. 2012;67:395–405. doi: 10.1093/gerona/glr223 22219514 PMC3309876

[27] Terry DF, Wilcox MA, McCormick MA, Perls TT Cardiovascular disease delay in centenarian offspring. J Gerontol A Biol Sci Med Sci. 2004;59:385–389. doi: 10.1093/gerona/59.4.m385 15071083

[28] Ismail K, Nussbaum L, Sebastiani P, Andersen S, Perls T, Barzilai N, et al. Compression of morbidity is observed across cohorts with exceptional longevity. J Am Geriatr Soc. 2016;64(8):1583–1591. doi: 10.1111/jgs.14222 27377170 PMC4988893

[29] Engberg H, Christensen K, Andersen-Ranberg K, Jeune B Cohort changes in cognitive function among Danish centenarians. A comparative study of 2 birth cohorts born in 1895 and 1905. Dement Geriatr Cogn Disord. 2008;26(2):153–160. doi: 10.1159/000149819 18679030 PMC2731155

[30] Engberg H, Oksuzyan A, Jeune B, Vaupel JW, Christensen K Centenarians–a useful model for healthy aging? A 29-year follow-up of hospitalizations among 40,000 Danes born in 1905. Aging Cell. 2009;8:270–276. doi: 10.1111/j.1474-9726.2009.00474.x 19627266 PMC2774420

[31] Ailshire JA, Beltrán-Sánchez H, Crimmins EM Becoming centenarians: disease and functioning trajectories of older US Adults as they survive to 100. J Gerontol A Biol Sci Med Sci. 2015;70:193–201. doi: 10.1093/gerona/glu124 25136001 PMC4311187

[32] Christensen K, McGue M, Petersen I, Jeune B, Vaupel JW Exceptional longevity does not result in excessive levels of disability. Proc Natl Acad Sci U S A. 2008;105:13274–13279. doi: 10.1073/pnas.0804931105 18711139 PMC2517602

