# Supplementary material for: Health Trajectories in Swedish Centenarians
Source: J Gerontol A Biol Sci Med Sci. 2020 Jun 22;76(1):157–63. doi: 10.1093/gerona/glaa152 (PMC7756707; doi:10.1093/gerona/glaa152)
Supplement: glaa152_suppl_Supplementary_Tables-and-Figures [file glaa152_suppl_supplementary_tables-and-figures.docx]

**Supplementary tables and figures**

**Table S1** List of the most common chronic diseases used in the study grouped following the International Classification of Diseases**−**10^th^ Revision

| **Disease groups** | **Individual diseases** | **ICD−10 or diagnosis criteria** |
| --- | --- | --- |
| Anaemia | Anaemia | Haemoglobin <13 g/dL in men and <12 g/dL in women |
| Cardiovascular diseases | Chronic rheumatic heart disease  Heart failure  Atrial fibrillation  Hypertension  Ischemic heart disease  Cerebrovascular diseases  Cardiomyopathy | I05**−**09  I50  I47  I10−15  I22−25  I60−69  I42 |
| Digestive disorders | Intestinal diverticula  Liver cirrhosis  Functional digestive disorders  Cholelithiasis | K57  K70−77  K59  K80 |
| Endocrine diseases | Thyroid dysfunction  Diabetes | E00−07  E10−14 |
| Malignancy | Malignant neoplasms | C00−97 |
| Neuropsychiatric diseases | Depression  Dementia  Schizophrenia  Alcohol dependence syndrome | F7; F10−19; F22; F29; F32; F34; F38; F43; F45; F48  DSM-III_R  F20−21  Z72 |
| Musculoskeletal diseases | Osteoporosis  Osteoarthrosis  Rheumatoid arthritis  Hip fraction  Crystal arthropathies  Polymyalgia rheumatica | M80−81  M15  M05−09  S72  M10  M35.3 |
| Neurosensorial diseases | Deafness  Visual impairments  Migraine  Peripheral neuropathies  Parkinson disease  Epilepsy | Being unable to hear the interviewer’s voice  Being blind or almost blind  G43  G50−73  G20  G40 |
| Respiratory diseases | Chronic obstructive pulmonary diseases  Pneumoconioses | J40−47  J60−66 |
| Urological disorders | Renal calculosis  Prostate hypertrophy  Renal failure | N20  N40  N18 |

**Table S2** Baseline characteristics of centenarians and non-centenarians after excluding participants <73 years old and >100.

|  | Non-centenarians  N=3,022 | Centenarians  N=196 | P value |
| --- | --- | --- | --- |
| Age | 83.4±5.4 | 88.7±6.4 | 0.001 |
| Female sex | 73.3% | 91.3% | <0.001 |
| Education level |  |  |  |
| *Elementary* | 42.9% | 39.6% | 0.229 |
| *High school* | 46.2% | 52.1% |  |
| *University or above* | 10.9% | 8.3% |  |
| Marital status |  |  |  |
| *Single* | 18.1% | 23.0% | <0.001 |
| *Widowed/divorced* | 54.8% | 63.8% |  |
| *Married/partnered* | 27.1% | 13.3% |  |
| Living situation |  |  |  |
| *Alone* | 61.4% | 76.5% | <0.001 |
| *With someone* | 25.9% | 13.3% |  |
| *In institution* | 12.8% | 10.2% |  |
| Number of chronic diseases | 3.7±2.2 | 3.4±2.1 | 0.122 |
| Number of Impaired ADLs | 0.8±1.5 | 0.6±1.2 | 0.020 |
| MMSE | 24.6±6.9 | 25.6±5.7 | 0.047 |
| Survival time | 6.8 ± 4.9 | 12.9 ± 6.5 | <0.001 |

Numbers are means ± standard deviations for continuous variables and percentages for categorical variables.

Abbreviations: ADL, Activities of Daily Living; MMSE, Mini Mental State Examination.

**Table S3** Baseline prevalence of the group of chronic conditions included in the study

|  |  |  |  |  |  |  |  |  |  |  |  |  |
| --- | --- | --- | --- | --- | --- | --- | --- | --- | --- | --- | --- | --- |
|  | **Non-centenarians** | | | | | | **Centenarians** | | | | | |
| **Chronic condition** | **All** | **60s** | **70s** | **80s** | **90s** | **100+** | **All** | **60s** | **70s** | **80s** | **90s** | **100+** |
| Anaemia | **15.8%** | 6.8% | 13.3% | 16.6% | 23.8% | n/a | **20.7%** | n/a | 8.3% | 9.8% | 25.2% | 38.5% |
| Cardiovascular diseases | **73.0%** | 70.8% | 70.5% | 74.0% | 76.5% | n/a | **68.5%** | n/a | 33.3% | 62.3% | 79.3% | 69.2% |
| Digestive disorders | **16.6%** | 9.3% | 16.3% | 15.2% | 24.7% | n/a | **20.3%** | n/a | 8.3% | 11.5% | 18.9% | 57.7% |
| Endocrine diseases | **18.0%** | 22.4% | 17.5% | 17.3% | 18.7% | n/a | 14.9% | n/a | 0% | 13.1% | 18.9% | 15.4% |
| Malignancy | **18.4%** | 10.7% | 21.4% | 18.6% | 15.3% | n/a | **8.1%** | n/a | 16.7% | 11.5% | 6.3% | 0% |
| Neuropsychiatric diseases | **23.1%** | 14.2% | 16.5% | 22.6% | 42.7% | n/a | **25.7%** | n/a | 4.2% | 3.3% | 31.5% | 73.1% |
| Musculoskeletal diseases | **33.9%** | 16.0% | 32.1% | 37.2% | 37.6% | n/a | **34.7%** | n/a | 20.8% | 37.7% | 36.9% | 30.8% |
| Neurosensorial diseases | **34.6%** | 28.8% | 33.0% | 30.0% | 49.7% | n/a | **50.9%** | n/a | 4.2% | 37.7% | 64.0% | 69.2% |
| Respiratory diseases | **8.3%** | 8.2% | 10.1% | 7.4% | 6.9% | n/a | **4.5%** | n/a | 0% | 3.3% | 7.2% | 0% |
| Urological disorders | **30.1%** | 16.7% | 29.2% | 28.3% | 43.6% | n/a | **43.2%** | n/a | 4.2% | 37.7% | 56.8% | 34.6% |

n/a information not available

**Table S4.** Estimated mean and 95% confidence intervals (CI) of chronic disease number, activities of daily living (ADL) impairments and mini mental examination score (MMSE)

| Age | N. chronic diseases  Mean (95% CI) | | ADL impairments  Mean (95% CI) | | MMSE score  Mean (95% CI) | |
| --- | --- | --- | --- | --- | --- | --- |
|  | **Non-centenarians** | **Centenarians** | **Non-centenarians** | **Centenarians** | **Non-centenarians** | **Centenarians** |
| 75 | 2.9  (2.8;3.0) | 1.9  (1.5;2.4) | 0.2  (0.1;0.2) | 1.0  (0.5;1.6) | 28.4  (28.2;28.6) | 26.2  (24.2;28.3) |
| 80 | 3.5  (3.4;3.6) | 2.7  (2.3;3.0) | 0.2  (0.1;0.3) | 0.2  (-0.1;0.5) | 27.0  (26.8;27.2) | 28.3  (27.0;29.5) |
| 85 | 4.0  (3.9;4.1) | 3.4  (3.0;3.7) | 0.5  (0.4;0.5) | -0.2  (-0.4;-0.1) | 24.8  (24.6;25.1) | 28.5  (27.6;29.5) |
| 90 | 4.6  (4.5;4.7) | 4.1  (3.8;4.3) | 0.8  (0.8;0.9) | -0.1  (0.3;0.0) | 22.0  (21.7;22.3) | 27.0  (26.0;27.9) |
| 95 | 5.1  (5.0;5.3) | 4.8  (4.5;5.0) | 1.4  (1.3;1.5) | 0.5  (0.3;0.7) | 18.4  (17.9;18.8) | 23.7  (22.8;24.5) |
| 100 | - | 5.5  (5.2;5.8) | - | 1.6  (1.3;1.9) | - | 18.6  (17.4;19.7) |
| 105 | - | 6.2  (5.8;6.6) | - | 3.2  (2.7;3.7) | - | 11.7  (9.6;13.7) |

*The table reports estimated means for representative ages (at 5-year intervals).*
